# Tail Resorption During Metamorphosis in *Xenopus* Tadpoles

**DOI:** 10.3389/fendo.2019.00143

**Published:** 2019-03-14

**Authors:** Yoshio Yaoita

**Affiliations:** Division of Embryology, Amphibian Research Center, Hiroshima University, Higashihiroshima, Japan

**Keywords:** tail resorption, *Xenopus*, metamorphosis, amphibian, thyroid hormone, thyroid hormone receptor, deiodinase, extracellular matrix

## Abstract

Tail resorption in anuran tadpoles is one of the most physically and physiologically notable phenomena in developmental biology. A tail that is over twice as long as the tadpole trunk is absorbed within several days, while concurrently the tadpole's locomotive function is continuously managed during the transition of the driving force from the tail to hindlimbs. Elaborate regulation is necessary to accomplish this locomotive switch. Tadpole's hindlimbs must develop from the limb-bud size to the mature size and the nervous system must be arranged to control movement before the tail is degenerated. The order of the development and growth of hindlimbs and the regression of the tail are regulated by the increasing levels of thyroid hormones (THs), the intracellular metabolism of THs, the expression levels of TH receptors, the expression of several effector genes, and other factors that can modulate TH signaling. The tail degeneration that is induced by the TH surge occurs through two mechanisms, direct TH-responsive cell death (suicide) and cell death caused by the degradation of the extracellular matrix and a loss of cellular anchorage (murder). These pathways lead to the collapse of the notochord, the contraction of surviving slow muscles, and, ultimately, the loss of the tail. In this review, I focus on the differential TH sensitivity of the tail and hindlimbs and the mechanism of tail resorption during *Xenopus* metamorphosis.

## Introduction

Metamorphosis occurs in most animal phyla and accompanies the concomitant morphological, ecological, and physiological changes. In the case of marine invertebrates, a larva acts mostly as a drifting or free-swimming creature in the ocean and thereby extends its habitat distribution and seeks out the optimal location for its survival, growth, and propagation, whereas the adult becomes a sessile animal or a burrower in the sea bottom after metamorphosis (1). Early Cambrian fossil records show that planktotrophic larvae metamorphosed into filter-feeding sedentary juveniles (2), which demonstrates the ancient origin and importance of metamorphosis in evolution.

An amphibian tadpole undergoes thyroid hormone (TH)-dependent metamorphosis from an aquatic to a terrestrial animal (3). TH-dependent metamorphosis is also reported in sea urchin (4), amphioxus (5), and flounder (6) to alter the lifestyle from that of a planktotrophic or free-swimming larva to that of a sessile or benthic adult. The developmental profile of gene expression in the rodent brain during the first 3 postnatal weeks resembles the corresponding profile in the *Xenopus* brain during the metamorphosis climax, which strongly suggests that the mammalian brain undergoes TH-dependent metamorphosis to adapt to the open-air environment after aquatic (amniotic) life, similar to anuran metamorphosis (7). In amniotes, TH-dependent metamorphosis might have evolved for adapting to environmental change during the rapid transition from amniotic to terrestrial life (8).

Anuran metamorphosis is characterized by the resorption of larva-specific organs (a tail and gills), the development of adult-specific organs (limbs), and the transformation of organs (brain, liver, intestine, pancreas, skin, etc.) from larval to adult type. Tail resorption is a particularly drastic change that occurs during the climax of anuran metamorphosis (9), and the phenomenon has attracted much scientific attention since the nineteenth century. A historical overview of the anatomical, physiological, and biochemical studies on tail resorption is presented elsewhere (10).

Tadpoles prepare for the smooth locomotive transition from fish-like swimming using a tail to frog-like swimming using hindlimbs by developing hindlimbs and arranging the nervous system to enable their powerful and accurately controlled movement (11), while the tail concurrently works as a constant driving force till the regression starts. This preparation is implemented by regulating blood TH levels. Whereas, hindlimbs can respond to low levels of THs by developing and growing, a tail cannot. Conversely, the tail responds to high levels of THs during the metamorphosis climax and initiates the death of muscle cells and the degeneration of the notochord, which lead to tail resorption. In this review, I introduce the models proposed to explain the differential TH sensitivity of the tail and hindlimbs and the mechanism of tail resorption.

## Differential TH Sensitivity of the Tail and Hindlimbs

### Developmental Regulation of THs and TH Receptors (TRs)

**Figure 1:**
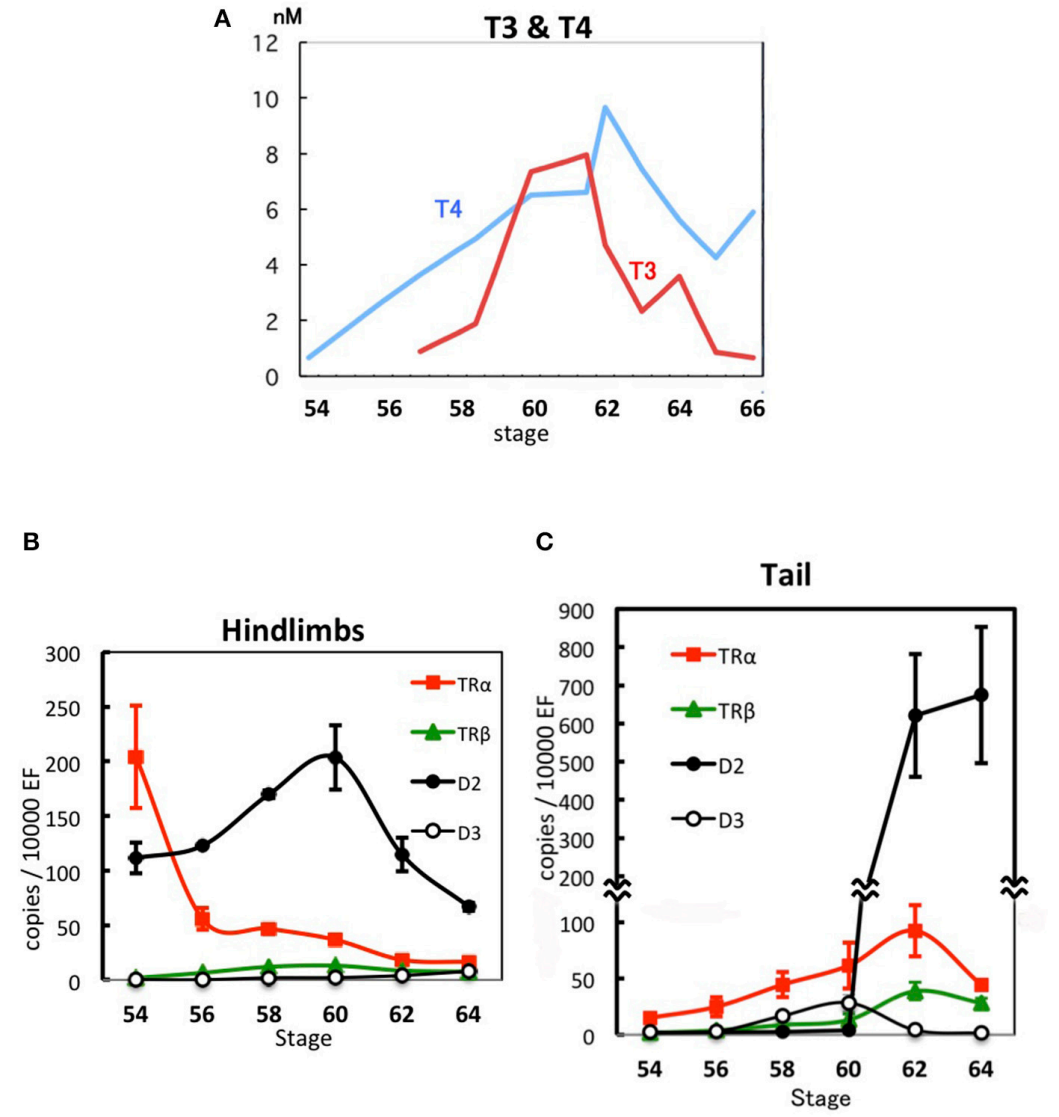
Developmental regulation of THs and gene expression during *X. laevis* metamorphosis. **(A)** TH levels in plasma (17). **(B)** Expression levels of *TR*α, *TR*β, *D2*, and *D3* mRNAs in hindlimbs (18). **(C)** Expression levels of *TR*α, *TR*β, *D2*, and *D3* mRNAs in the tadpole tail (18). Data are shown as means ± SE. *EF, elongation factor 1*α.

TH binds to a heterodimeric receptor—composed of TR and 9-cis retinoic acid receptor—that inhibits and activates transcription from promoters containing the TH-response element (TRE) in the absence and presence of TH, respectively (12, 13). Vertebrates express two TR subtypes, TRα and TRβ, and hindlimb growth is inhibited by unliganded TRα (14–16) before endogenous TH secretion from the thyroid gland begins, i.e., before Nieuwkoop and Faber (NF) stage 54 of *Xenopus laevis* (17). The plasma level of thyroxine (T4), a low-activity TH precursor, slowly increases from NF stage 54 (0.66 nM) to NF stage 62 (9.7 nM), and this is accompanied by hindlimb growth (Figure 1A). Conversely, 3,5,3′-triiodothyronine (T3), a highly active TH derived from T4, surges abruptly at NF stage 58, the beginning of the metamorphosis climax, and peaks at NF stages 61–62 (7.9 nM), when the tail starts shortening. T3 shows 4 to 7-fold-higher binding affinity for TR than T4 (19, 20). The tail starts regressing only after the hindlimbs have grown adequately and move cooperatively to enable swimming, which indicates that hindlimbs can respond to substantially lower levels of THs than the tail.

As THs circulate in the bloodstream throughout the body and all organs and tissues are exposed to the same concentrations of THs, when and how each organ or tissue orchestrates the induced transformation depends on their sensitivity and responsivity to these metamorphosis inducers, and this is expected to be reflected by the developmental expression of the genes involved in TH signaling in the transforming organs. The expression level of *TR*α mRNA in the hindlimbs of *X. laevis* is high at NF stage 54 and decreases up to NF stage 62, whereas *TR*β mRNA is expressed at very low levels throughout metamorphosis (Figure 1B). In contrast, *TR*α mRNA level in the tail rises gradually from NF stage 54 to 62 and then decreases, whereas *TR*β mRNA is expressed at very low levels and increases starting from NF stage 62 (18, 21–23) (Figure 1C). Therefore, the decreasing *TR*α mRNA expression in the hindlimbs and the increasing *TR*α mRNA expression in the tail intersect during development around NF stage 58 when the level of active T3 rises sharply, which suggests that the TH sensitivity of organs depends on the TRα expression level. This is supported by a report that the TH sensitivity of tail tips increases steadily with development from NF stage 38–58 in organ culture, as evidenced by a shortening lag period before the onset of regression and an increased rate of regression (24).

### Enhancement of TH Sensitivity by TR Overexpression in the Tail

Somatic gene transfer performed using electroporation enables the introduction of exogenous DNA into many tail muscle cells. Moreover, treatment with an inhibitor of TH synthesis, methimazole, stops tadpole development and hindlimb growth at NF stage 54. If methimazole-treated tadpoles are immersed in low-concentration (0.3–1 nM) T4 and T3 solutions, hindlimb buds can grow within several days in a TH-dose-dependent manner, whereas the tail cannot regress. However, when a *TR*-expression construct is introduced (together with a reporter gene) into the tail muscle cells of methimazole-treated tadpoles, tail cells respond to low levels of THs and die over a time course similar to that of hindlimb growth; therefore, *TR* mRNA overexpression confers responsiveness to low levels of T3 and T4 on tail muscle cells (18). The disappearance of tail muscle cells induced by T4 treatment is delayed compared with the disappearance after T3 treatment, which implies that T4 is converted to the active form, T3, by the induction of type 2 iodothyronine deiodinase (D2). Furthermore, the D2 inhibitor iopanoic acid impairs the death of *TR*-overexpressing tail muscle cells as well as the growth of hindlimbs at a low level of T4 but not T3, which supports a role for D2 in the response to low T4 levels (18).

### Developmental Expression of the Gene *D2* and Its Regulation by THs

D2 activity and mRNA levels are the highest in a given tissue at the time of the tissue's major transformation (25). *D2* mRNA is expressed at a high level in growing hindlimbs at NF stage 54 and increases up to NF stage 60, whereas the mRNA is present at a low level in the tail and is abruptly elevated to an extremely high level at NF stage 62, when tail regression starts (18, 26) (Figures 1B,C). The *D2* mRNA level in hindlimbs is reduced to one-fourth of the control level after 1 month of methimazole treatment of NF stage 54 tadpoles, which means that only a small fraction of the *D2* mRNA in untreated tadpoles is expressed in hindlimbs in the absence of THs and the major fraction is expressed in a TH-dependent manner. Treatment with a low concentration of T4 induces *D2* mRNA in 4 days in the *TR*α-overexpressing tail muscle cells of methimazole-treated tadpoles, and the levels of *D2* mRNA and *TR*α mRNA show a close correlation (18).

*D2* mRNA is induced in 8 h by T3 in the tail myoblastic cell line XLT-15, and the induction is only partially abrogated by the protein synthesis inhibitor cycloheximide, which indicates that *D2* is a direct TH-response gene (18). This is supported by the presence of a functional TRE at similar positions within 600 bp of a highly conserved region in *X. laevis* D2.L and D2.S genes and *Xenopus tropicalis* D2 gene; the TREs are located 1–2 kb upstream of the TATA box. The *D2* TREs exhibit lower affinity (i.e., they are weak TREs) for TR *in vitro* and lower homology to the TRE consensus sequence than the TRE of another direct TH-response gene, *TR*β. Moreover, *D2* mRNA expression is activated 4-fold more by a low level of T3 in *TR*-transfected cultured cells than in vector-transfected cells, whereas the *TR*β mRNA level shows no difference between *TR*- and vector-transfected cells. A larger amount of the *TR*-expression construct is required for the *D2* TRE to mediate sufficient TH signaling in the oocyte system than for *TR*β TRE-mediated signaling (18).

### Low Sensitivity to T4 in the Tail of Young Tadpoles

**Figure 2:**
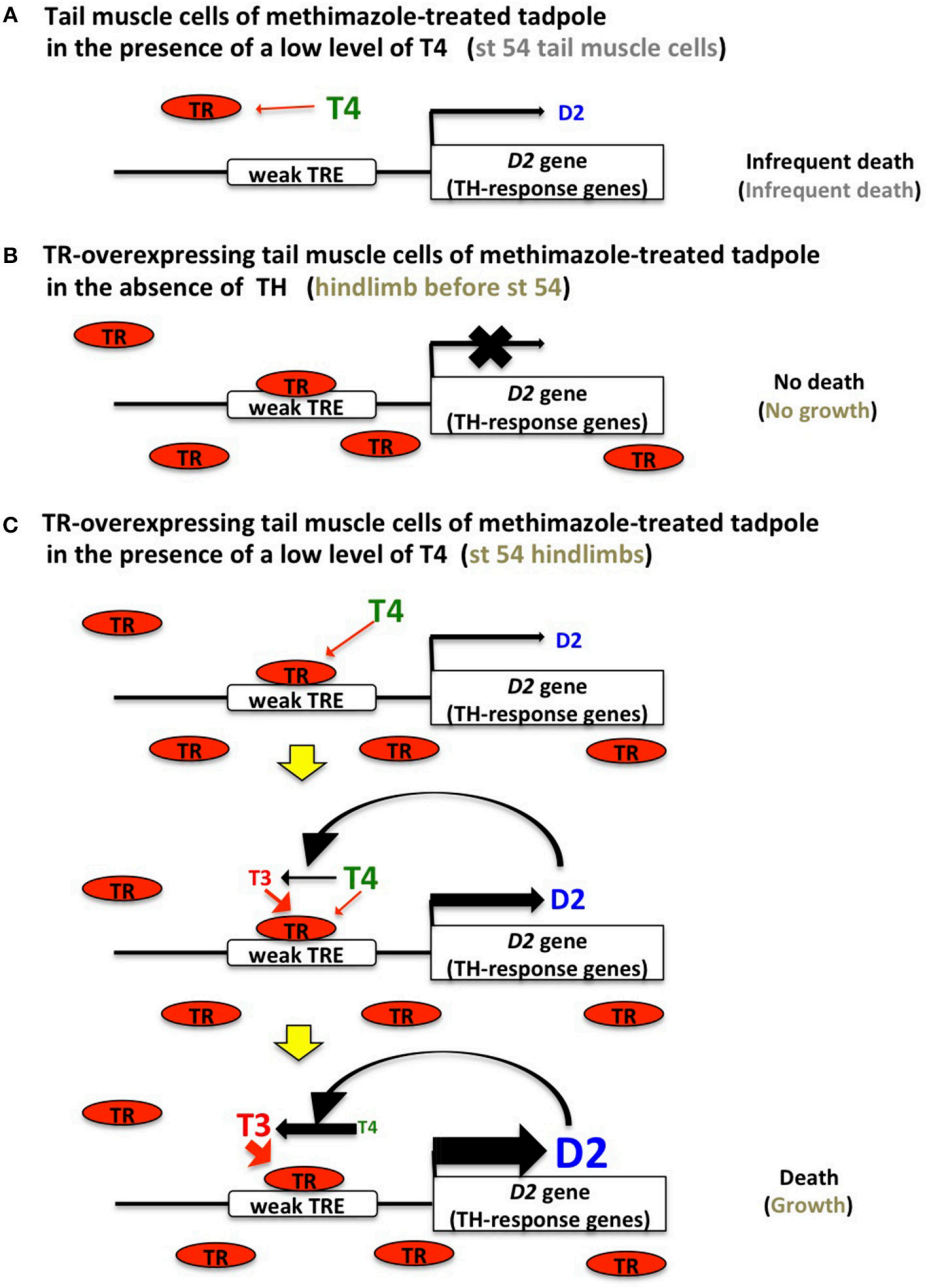
TH sensitivity and response in tail muscle and hindlimb cells. **(A)** TR expression is too low to bind to the low-affinity TREs (weak TRE) of *D2* and TH-response effector genes in tail muscle cells of methimazole-treated tadpoles and stage 54 tadpoles, which results in a leaky expression of these genes and induces cell death infrequently. **(B)** Abundant and unliganded TR binds to TREs in TR-transfected tail muscle cells of methimazole-treated tadpoles and stage 54 tadpole hindlimbs and inhibits cell death and growth, respectively, by repressing the expression of TH-response genes. **(C)** Once a low level of T4 is recruited to the TR on the weak TRE of the *D2* gene, D2 protein is gradually synthesized and converts T4 to T3, which stimulates *D2* expression more efficiently. This positive feedback loop drives the expression of *D2* and TH-response genes and TH activation, leading to cell death in TR-overexpressing tail muscle cells of methimazole-treated tadpoles in the presence of a low T4 level and growth in stage 54 tadpole hindlimbs.

Low levels of *TR* mRNAs are produced in the tadpole tail at NF stage 54. In vector-transfected tadpoles treated with methimazole for several days, a small fraction of the transfected tail muscle cells disappears even in the absence of TH, compared with TR-overexpressing muscle cells in the same condition. A similar cell-death tendency of vector-transfected cells is observed in the presence of 0.3–1 nM T4 (18). If a low level of endogenous TR binds to the weak TREs of TH-response genes, including *D2* and several effector genes (27, 28), TR should repress the expression of TH-response genes causing cell death in the absence of T4, as seen in TR-overexpressing muscle cells. The similar death pattern exhibited by vector-transfected tail muscle cells in the presence of 0, 0.3, and 1 nM T4 could be ascribed to the expression of endogenous TR at a level that is too low to form a stable complex with the weak TREs of TH-response genes (*D2* and other effector genes), which then allows the leaky expression of the genes to induce the death of a small fraction of cells. This view suggests that endogenous unliganded TR cannot completely inhibit the metamorphic change of tail cells. The expression of endogenous TR at insufficient levels to inhibit TH-target genes in young tadpoles may be similar to the inadequate TR levels to elicit the maximal response to TH. The latter is supported by the report that a desert frog, which features the shortest larval period, expresses elevated levels of *TR*α mRNA throughout development and exhibits accelerated expression kinetics of TH-response genes under exogenous TH treatment. Furthermore, overexpression of *TR*α increases the rate of tail muscle cell death in response to TH (29). The death of a small fraction of tail muscle cells should be observed in normal NF stage 54 tadpoles (Figure 2A); the dead cells might be replenished through the cell division of myoblasts during development.

In the hindlimbs of *TR*α-knockout tadpoles, TH-response effector genes are no longer repressed by unliganded TR due to the complete loss of TRα and their expression induces precocious development before NF stage 54 (14–16). The hindlimbs show reduced responsiveness to 10 nM T3 at NF stages 53–55 (30), suggesting a low sensitivity to TH. The growth rate of hindlimbs in *TR*α-knockout tadpoles is higher at NF stages 50–54 (in the absence of THs), similar to NF stages 54–56, and lower thereafter than the rates in wild-type and *TR*β-knockout hindlimbs, which catch up to the *TR*α-knockout hindlimbs in size at NF stage 58 (16). Wild-type and *TR*β-knockout hindlimbs show no difference in morphology or size, and thus TRβ plays only a minor role in the growth and development of hindlimbs.

When a *TR*-expression construct is introduced into the tail muscle cells of methimazole-treated tadpoles, the overexpressed TR binds to the weak TREs of *D2* and other TH-response effector genes and represses their expression because of the absence of THs (Figure 2B). The slow growth of tail muscle cells that is obscured by cell death becomes apparent following the complete inhibition of cell death (18). Given that *TR*α mRNA is abundant in the hindlimbs of wild-type tadpoles before NF stage 54, inhibition of TH-response effector genes by TR in the absence of THs constrains growth and development.

### High Sensitivity to T4 in TR-Overexpressing Tail Muscle Cells or Hindlimbs of Young Tadpoles

The organs that express TR abundantly can respond to a low level of T3 as a result of the stable interaction between liganded TR and the weak TREs of effector genes in the absence of *D2* mRNA induction and TH activation; conversely, the organs that express TR at low levels cannot drive the response to a low level of T3, because the high TR expression level is required to increase the occupancy at weak TREs, independent of T3 concentration.

A positive-feedback model involving the upregulation of *D2* and the conversion of T4 to T3 by D2 enzymatic activity in the presence of low levels of T4 has been proposed to explain the organ sensitivity to T4 (18) (Figure 2C). Abundant TR forms a stable complex with the weak TREs of TH-response genes, including *D2* and effector genes in the hindlimbs of NF stage 54 tadpoles and TR-overexpressing tail muscle cells of methimazole-treated tadpoles, binds to a low level of T4 (0.3–1 nM) transiently and weakly, and stimulates the *D2* promoter to induce the production of a small amount of the D2 enzyme. Following this subtle induction of D2 production, T4 is converted to T3 by the enzyme, and the generated T3 then binds to the TR recruited on the TREs of *D2* and other effector genes more robustly and efficiently and activates *D2* transcription leading to further conversion of T4. This process might be promoted by the stimulated expression of *TR*β that has a high affinity TRE, although *TR*β mRNA is upregulated weakly by 1 nM T4 in 4 days in TR-overexpressing tail muscle cells (18). This positive-feedback loop operating under abundant TR expression amplifies TH signaling through *D2* stimulation and TH activation to enable a response to a low level of T4 earlier than in other organs, and this then establishes the metamorphic changes such as limb growth within several days. During the positive-feedback process, TH-response effector genes harboring weak TREs are also activated to drive the growth in hindlimbs or the cell death in the tail.

### Developmental Gene Expression and Function of Type 3 Iodothyronine Deiodinase (D3)

Differential sensitivity of organs and tissues to THs is regulated by multiple molecular mechanisms (31). Whereas D2 converts T4 to T3 and thereby leads to TH activation, D3 inactivates T3 and T4. *D3* mRNA expression level and D3 activity in the tail are low at NF stage 54, increase from NF stage 58 to 60–61, and decrease thereafter (18, 32–34) (Figure 1C). D3 activity appears to inhibit the expression of TH-response effector genes before NF stage 61 by reducing the TH concentration in the tail (35). Their prominent and concurrent expression is induced at NF stage 62 after the downregulation of *D3* mRNA (28). The *D3* mRNA level is increased in Meckel's cartilage immediately before the end of its proliferation during the late metamorphosis climax, and, similarly, in late limb development. The *D3* mRNA level typically declines immediately before a tissue's metamorphic change, which enables the tissue to respond to THs, or increases immediately before the completion of a tissue's response to TH. Thus, D3 expression is involved in the elaborate regulation of the TH response of organs (36). Because tail tips can respond to lower levels of THs in organ cultures during developmental progression from NF stage 38–58 (24), D3 might reduce the TH responsivity of the tail at NF stages 59–61 (31).

### Developmental Gene Expression and Function of TH Transporters and Cytosolic TH-Binding Proteins

MCT8, MCT10, OATP1c1, and LAT1 are TH transporters, and their mRNAs are upregulated when an organ undergoes TH-dependent metamorphic changes (37). However, *OATP1c1* mRNA is expressed at low levels in hindlimbs and the tail during metamorphosis (38). *LAT1* mRNA is increased at a single stage of development in the hindlimbs and tail, NF stage 54 and stage 62, respectively (37). Intriguingly, *MCT8* mRNA expression in hindlimbs is high at NF stage 54, decreases from NF stage 58–60, and then remains at a low level. However, in the tail, the mRNA is expressed at NF stage 54 at a low level and then increases from NF stage 62–64, suggesting the mRNA level reflects the TH sensitivity of each organ. In humans, an *MCT8* mutation causes Allan-Herndon-Dudley syndrome, which is characterized by X-linked mental retardation and markedly elevated serum T3 (39, 40). Skin fibroblasts of patients with the syndrome show decreased T4 and T3 uptake, which indicates that MCT8 is a major TH transporter expressed in dermal fibroblasts (41). However, *MCT8* overexpression fails to promote TH-induced death of tail muscle cells in response to 2 nM T3, whereas *LAT1* overexpression increases the rate of tail cell death (38).

A previous study analyzed the mRNA expression during metamorphosis of three cytosolic TH-binding proteins, aldehyde dehydrogenase 1, pyruvate kinase subtype M2, and μ-crystallin (CRYM) (38). *CRYM* mRNA expression declines from NF stage 54 to 66 in hindlimbs, but increases from NF stage 63 onward in the tail, thus exhibiting an expression pattern similar to that of *MCT8* mRNA. The affinity constant of T3 binding to CRYM is approximately 2 nM, and T3 concentration in tissues is reduced in *CRYM*-knockout mice without alternations of peripheral T3 action, suggesting that TH is retained inside cells by CRYM. Interestingly, a *CRYM* mutation has been reported in two families with hereditary deafness (42). Lastly, overexpression of *CRYM* or the pyruvate kinase subtype M2 gene, like *LAT1* overexpression, results in enhanced tail muscle cell death in the presence of 2 nM T3, and co-overexpression of *MCT8* and *CRYM* produces a synergistic effect on cell death (38).

## Mechanism of Tail Resorption

### Murder Model

When tailfin explants are incubated in a TH-containing solution, collagenase activity and loss of tissue collagen are induced together with the progression of tailfin resorption (43). The regression program stops in the tail amputated from a tadpole that has been treated with 100 nM T3 for 48 h if a protein synthesis inhibitor is added within 24 h of TH pretreatment, but if the tadpole is treated with TH for >48 h, the regression continues even if TH is removed or the protein synthesis inhibitor is added. These findings suggest that the genes involved in tail regression are activated during the 2 days of TH treatment (44). In this study, roughly 20 TH-upregulated genes were isolated by employing a PCR-based subtractive-hybridization procedure using RNA isolated from the tails of NF stage 54 tadpoles treated with 100 nM T3 for 1–2 days (44). The mRNA levels of these genes increase developmentally in the tail during the normal metamorphosis climax, and the upregulated genes include not only a direct TH-response gene, *TR*β, but also *collagenase 3* (*MMP13*), *stromelysin-3* (*MMP11*), and *fibroblast activation protein* α (*FAP*α) (33). MMP13 cleaves types I, II, and III collagen and gelatin (45) and belongs to the matrix metalloproteinase (MMP) family of enzymes that degrade extracellular matrix (ECM) proteins between cells (46). FAPα is a homodimeric integral-membrane gelatinase belonging to the serine-protease family. Subsequent work revealed that the regressing tail during the metamorphosis climax expresses several MMP genes, *MMP18* (*collagenase 4*) (47), *MMP2* (*gelatinase A*) (48), *MMP9TH* (27), and *MMP14* (*membrane-type 1 MMP*) (49), which are concomitantly upregulated in response to the TH surge at NF stage 62 when the tail begins to regress (28).

The proteolytic-enzyme mRNAs accumulate at high levels at NF stage 63 in the proliferative fibroblasts of the tail. These cells line and surround the notochord sheath or lie beneath the epidermal lamella and start invading the notochord or their neighboring epidermal collagen lamella, respectively, at late NF stage 63 and early NF stage 64. This fibroblast invasion is suggested to participate in notochord collapse and tail regression. The murder model is proposed based on these observations (31). The fibroblasts around the notochord and under the epidermal lamella start producing ECM-degrading proteases at NF stage 62 in response to the peak level of T3 and migrate to the epidermal lamella, notochord sheath, and the basal lamina between muscle cells. These cells secrete ECM-degrading enzymes that dissolve the basal lamina, and widespread ECM degradation results in the loss of cellular attachment to the ECM, elimination of anchorage, and death of muscle cells.

The tail muscle is mostly composed of fast muscle, whereas the peripheral muscle fibers are slow-muscle fibers. During ECM cleavage and digestion in the tail by MMPs at around NF stage 62, the bulk of fast muscle disappears preferentially. Subsequently, during notochord degeneration and collapse at NF stages 62–65, rapid tail shortening is driven by the contraction of four muscle cords comprising two dorsal and two ventral parallel rows of slow-muscle bundles that run along the tail's length (50).

### Suicide Model

Muscle cells die in the resorbing tail during the metamorphosis climax and are fragmented into membrane-bounded muscle pieces, engulfed, and digested by macrophages, which is typical of apoptosis (51). To facilitate analysis of tail resorption, myoblastic cell lines were established from the *X. laevis* NF stage 57 tadpole tail. In these cells, apoptosis occurs in response to physiological TH concentrations, as indicated by positive TUNEL (terminal deoxynucleotidyl transferase-mediated dUTP nick-end labeling) reaction and internucleosomal DNA cleavage. TH-triggered death of the myoblastic cells is not stimulated by the addition of conditioned medium collected from a cell line incubated with T3 for 2 days, which suggests that cell death is not mediated by a paracrine mechanism, such as a mechanism involving ECM-degrading enzymes that dismantle cell anchorage or secreted factors that induce apoptosis. These findings have engendered the suicide model, which posits that tail muscle cells respond cell-autonomously to TH by undergoing apoptosis, because the myoblastic cell line represents a homogenous population derived from a single cell of tail muscle and includes no fibroblasts (52).

TH induces *caspase 3* mRNA in one of the established cell lines, XLT-15, and the mRNA is also induced in the regressing tail during the metamorphosis climax. Moreover, the apoptosis of XLT-15 cells by TH is blocked by a Caspase 3 and 7 inhibitor (acetyl-Asp-Glu-Val-Asp-aldehyde). These results imply that cell death is triggered by the induction of *caspase 3* mRNA. However, *caspase 3* mRNA is not induced by TH in a subline of XLT-15, XLT-15-11, that also dies in response to TH, which indicates that the induction of *caspase 3* mRNA is not essential for cell death, although Caspase 3 might promote apoptosis and tail resorption. Furthermore, *caspase 1, 2*, 6, *7, 8, 9*, and *10* are not upregulated by TH in XLT-15-11 cells (53). The induction of these apoptosis-related genes is not necessary for the death of myoblastic cell lines, but the genes might functionally complement each other and coordinate with TH-induced apoptosis-promoting genes that are as yet unidentified. Although the pro-apoptotic genes *bax* and *bid* are suggested to be involved in tail resorption (54, 55), whether they are required for the death of tail cells remains to be determined.

### Death Switch From Suicide to Suicide and Murder at the Beginning of Tail Regression

In response to TH, the myoblastic cell line XLT-15 transcribes the mRNAs of *MMP9TH* and *FAP*α (28). *MMP9TH* is one of the duplicated genes of *MMP9*. *MMP9TH* expression is strongly induced by THs, whereas *MMP9* expression is not (27). MMP9TH exhibits gelatin-degrading activity like MMP9. MMP9 cleaves native type IV collagen, which is the major structural component in the basal lamina that underlies all epithelial sheets and tubes and encircles single muscle cells. In NF stage 63 *TR*β-knockout tadpoles, *MMP9TH* and *FAP*α expression levels are reduced to less than one-tenth in the posterior part of the tail, where almost all muscle flanks and the satellite cells between muscle cells disappear, compared with the corresponding levels in the tail of wild-type and *TR*α-knockout tadpoles at the same stage (16). These observations suggest that muscle cells and/or satellite cells synthesize *MMP9TH* and *FAP*α mRNAs in response to THs and thus dismantle the basal lamina surrounding individual muscle cells.

In a dominant-negative form of TR (DNTR), a mutation in the TH-binding domain compromises the TH-binding ability of the protein. DNTR binds to the TRE in the promoter of TH-response genes and represses their expression by associating with co-repressors, irrespective of the presence of TH (56, 57). Following co-transfection of a *DNTR*-expression construct and a reporter gene into XLT-15 cells, DNTR-overexpressing XLT-15 cells survive for 3 days in the presence of 10 nM T3, whereas half the vector-transfected control cells die. Given that expression of the exogenous reporter gene is detected in only a fraction of the cells, most of the cells respond to T3 and secrete MMP enzymes. Because DNTR overexpression cannot protect even *DNTR*-transfected cells against anchorage dissolution by ECM-degrading enzymes, DNTR inhibition of the death of the cultured cells indicates that DNTR blocks the TH signaling leading to cell-autonomous suicide, which agrees with the result of the experiment conducted using the conditioned medium mentioned above (58). The action of the ECM-degrading enzymes might be compromised by MMP inhibitors contained in the fetal calf serum included in culture medium (59).

**Figure 3:**
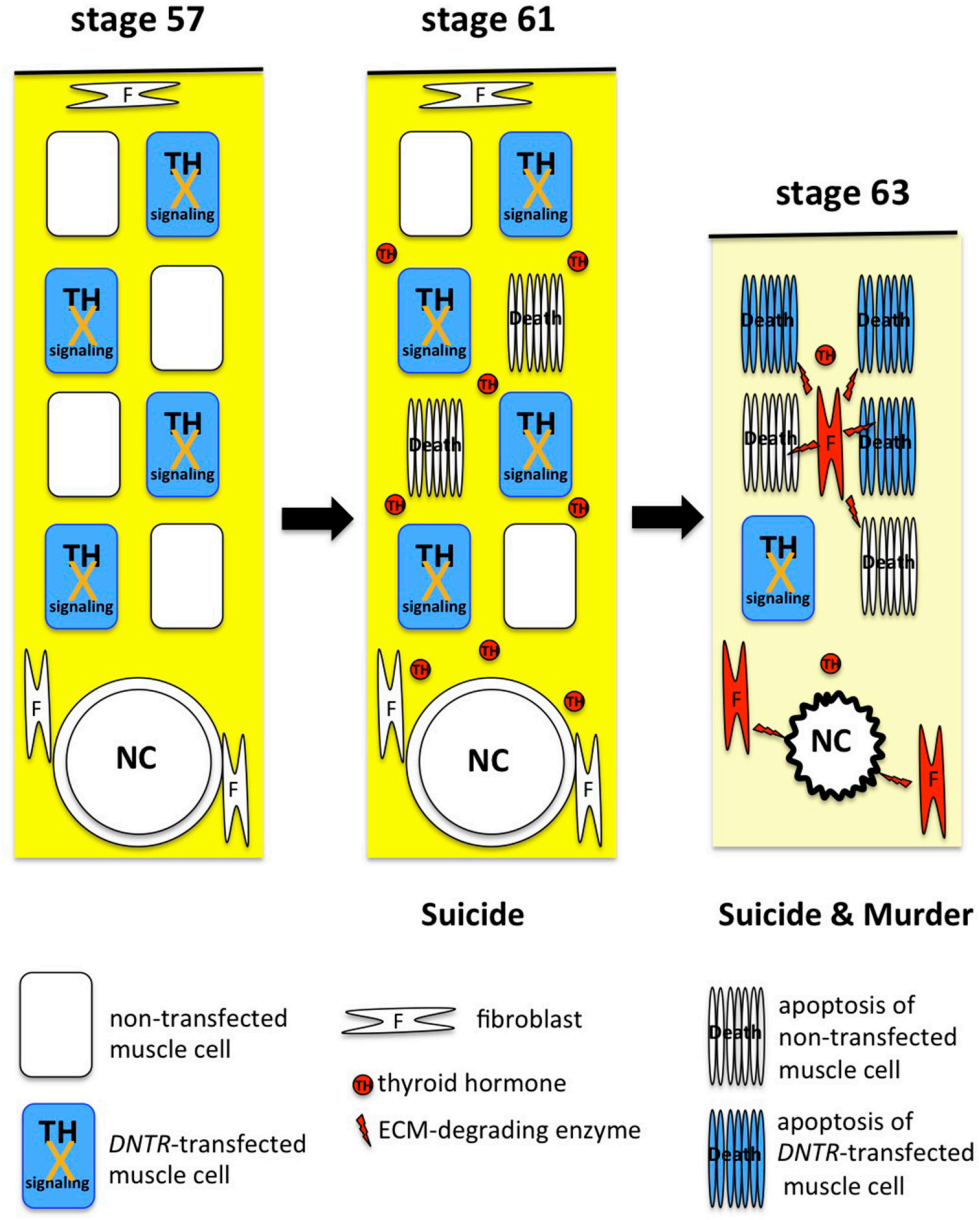
Suicide and murder model of tail resorption. TH induces muscle cells to commit suicide and drives fibroblasts (red) and muscle cells to produce and secrete ECM-degrading enzymes for dissolution of the ECM (yellow), apoptosis of muscle cells, and collapse of the notochord (NC).

A *DNTR*-expression construct has been introduced together with a reporter gene into muscle cells in the tail of live tadpoles to block TH signaling and analyze the effect of DNTR expression on cell death *in vivo*. According to the suicide model, DNTR overexpression represses the suicide process only in *DNTR*-transfected muscle cells and enables their survival in the presence of TH. Conversely, under the murder model, all *DNTR*-transfected cells and non-transfected cells are killed by the translated death-promoting proteins encoded by the TH-response genes that are induced in a majority of non-transfected muscle cells and fibroblasts. The blocking of TH signaling by DNTR overexpression almost completely inhibits muscle cell death until NF stage 61 (i.e., until immediately before tail regression), but does not protect *DNTR*-transfected cells after NF stage 62, when the genes for several ECM-degrading enzymes are upregulated drastically and concurrently and the tail starts regressing (28). Only 10% of *DNTR*-transfected cells survive after the metamorphosis climax, and some of the cells are retained even 3 weeks after metamorphosis, which indicates that DNTR overexpression inhibits cell death partially by attenuating cell death by suicide. These findings show that TH induces tail muscle cells to commit suicide before tail regression and to die through the suicide and murder mechanisms after the regression (58) (Figure 3).

### Immunological Rejection Model and Its Reexamination

*Xenopus laevis* juveniles are reported to reject tail skin grafts from syngeneic tadpoles, but trunk skin grafts become increasingly more acceptable with the progression of metamorphosis of the donor tadpoles. This tadpole skin rejection starts with the recognition of the tadpole-specific skin proteins Ouro1 and Ouro2 as non-self-proteins. Precocious tail degeneration is elicited before the climax when *ouro1* and *ouro2* are overexpressed using a heat shock-inducible promoter, whereas tail resorption during spontaneous metamorphosis is delayed in response to antisense *ouro* RNA-induced knockdown of *ouro1* and *ouro2* using the same promoter, which results in the generation of tailed froglets (60). These data suggest that larval organs such as the tail are eliminated during the metamorphosis climax in the tadpole through the recognition of larva-specific proteins as non-self-proteins by the immune system.

The knockouts of *ouro1* and *ouro2* were generated using the genomic-editing technique in *X. tropicalis* to reexamine the immunological rejection model. While *ouro1*-knockout tadpoles express no Ouro1 and express Ouro2 at a very low level, *ouro2* knockouts express no Ouro2, and express Ouro1 at a barely detectable level. However, the *ouro1*- and *ouro2*-knockout tadpoles undergo normal metamorphosis without any morphological differences from wild-type tadpoles, and the knockouts do not retain a tail after metamorphosis (61).

Athymic frogs were created by modifying *Foxn1*, a gene whose mutation in mice results in congenital loss of the thymus and mature T cells, including helper and CD8^+^ cytotoxic T cells, and in a severe combined immunodeficiency phenotype (62). Similarly, athymic frogs harbor no splenic CD8^+^ T cells, which are necessary for a cytotoxic reaction, and these frogs can accept major histocompatibility-disparate skin grafts. However, the tadpole tail disappears normally in these frogs during the metamorphosis climax without delay (61).

Although the skin grafts of tadpoles appear to be rejected reproducibly on the syngeneic frogs of *X. tropicalis* within 2 months as discussed above, the grafts survive for >150 days on syngeneic frogs treated with a TH-synthesis inhibitor (methimazole) for 1 month before the skin transplantation and continuously during the experiment. Because methimazole exhibits no immunosuppressive activity, the result indicates the possibility that the skin grafts become atrophic in response to the THs derived from the recipient frogs (63). This notion is supported by results demonstrating that the serum of wild-type *X. tropicalis* frogs contains 6.3 nM T4, which is comparable to the concentration in the *X. laevis* tadpole featuring a regressing tail during the metamorphosis climax (63). In addition, one of the TH-response genes, *TR*β, is expressed in the adult brain and liver at a level similar to the late phase of the metamorphosis climax (7). These findings show that the endogenous levels of THs circulating in a frog induce the degeneration of the syngeneic tadpole skin graft.

The results of the analyses on the *ouro*-knockouts, athymic tadpoles, and skin-graft transplantation are incompatible with the immunological rejection model. I cannot exclude the possibility that this discrepancy is because of the species difference between *X. laevis* and *X. tropicalis*, but it is also likely that the active apoptosis pathway in a regressing tail is impaired by the toxic effect of heat shock, leading to unphysiological results. If heat-shock treatment disrupts the progression of metamorphosis and the cell-death process, the use of other promoters and knockout experiments might be more appropriate for examining how metamorphosis is affected by the overexpression and reduced expression of a gene of interest, respectively.

### Tail Resorption in *TR*-Knockout Tadpoles

Wild-type and *TR*α-knockout tadpoles show no differences in developmental tail regression during the metamorphosis climax (14–16) in either histological or quantitative gene-expression analysis (16). However, the precocious development of hindlimbs is observed during premetamorphosis before TH secretion (14–16) and the expression levels of several brain genes are reduced at NF stage 61 in *TR*α-knockout tadpoles (7). The absence of TRα leads to *TR*β de-repression, accumulation, and subsequent recruitment to the TRE of *TR*β to repress its expression in the absence of THs (14), but is not enough to bind to the weak TREs. As the T3 level increases, *TR*β expression is immediately activated by the T3-bound TR recruited on the TRE to compensate for the lack of TRα and to express sufficient levels of TR for binding to the weak TREs of the TH-response effector genes. This augmentation of TH signaling induces cell death and, ultimately, the collapse of the notochord according to the regular timetable.

Tail regression, gill absorption, and olfactory-nerve shortening are markedly delayed in *TR*β-knockout tadpoles (16). This delay of tail regression is reproduced in *TR*β-knockdown tadpoles (64). Complete tail loss requires 3–4 weeks after the start of tail regression at NF stage 62 in *TR*β-knockout tadpoles, compared with 1 week in wild-type and *TR*α-knockout tadpoles, which demonstrates that TRβ plays a dominant role in tail resorption (16). *TR*β-knockout tadpoles retain a tail after their body undergoes metamorphosis, which make them appear as tailed frogs (16). As *TR*β-knockout tadpoles complete metamorphosis slowly over time, TRα expression may finally compensate for the loss of TRβ.

Although muscle cells disappear almost completely and the tail is shortened to three-quarters of the trunk length by 5 days after NF stage 62 in *TR*β-knockout tadpoles, the tail still harbors a healthy notochord, which runs along the tail's length and supports the structure. In contrast, the notochord is dissolved 1 day after NF stage 62 in wild-type and *TR*α-knockout tadpoles featuring a tail of similar size. Interestingly, the anterior part of the tail expresses a higher level of *MMP13* mRNA in NF stage 63 *TR*β-knockout tadpoles than in wild-type and *TR*α-knockout tadpoles at the same stage, although the expression of all *MMP* mRNAs examined is decreased in the distal part of the tail in *TR*β-knockout tadpoles. *MMP13* mRNA exhibits very strong expression in the notochord, notochord sheath, and fibroblasts around the notochord during the climax of spontaneous metamorphosis (31). However, the notochord shows no detectable degradation in *TR*β-knockout tadpoles despite the >2-fold-increased expression of *MMP13* mRNA, whose product exhibits collagenase activity after proteolytic processing. This observation suggests that the activation of MMPs, including MMP13, is attenuated and delayed in the absence of TRβ. To my knowledge, tailed frogs are created in response to treatment with a TH-synthesis inhibitor (50) and overexpression of transgenic *D3* (35), *prolactin* (65), and the gene encoding a dominant-negative form of SRC3 (a member of the steroid receptor coactivator family) (66), all of which inhibit the TH-signaling pathway. Thus, maximal TH signaling might be essential for the collapse of the notochord (50). *TR*β is a direct TH-response gene that is upregulated, and this culminates in the production of sufficient levels of TR through autoregulation for binding to the weak TREs of effector genes (22). One of these effector genes might encode an activator protein that converts a latent MMP into an active enzyme and thus helps dismantle the notochord. Once the notochord weakens, the tail begins shortening through the contraction of the four cords, as mentioned above (50).

## Perspectives

The TH-dependent anuran metamorphosis might be required for the quick transformation from an aquatic to a terrestrial form that enables anurans to escape from predators and hunt for prey readily and rapidly without interruption. The metamorphic change is repressed by unliganded TRα in the TH-sensitive organs before TH secretion, is induced by a gradual increase of T4 in the organs with abundant expression of TRα for the preparation to adapt to adult life, and occurs in almost all organs at the peak concentration of T3 to eliminate the larval organs and accomplish the transformation by the induction of TRβ. This prompt, coordinated, and systematic remodeling orchestrates appropriate and precise development for survival, which is executed by the differential expression of *TR*α mRNA at high and low levels in the hindlimbs and the tail of young tadpoles, respectively. The 5′-untranslated region of *TR*α mRNA contains several translational repressive elements that control TRα expression at low levels (67). However, the mechanism by which TRα expression is regulated at the transcriptional and translational levels in the hindlimbs and the tail remains to be investigated.

Tadpole tail resorption during metamorphosis has long been studied (10), and the apoptotic pathway involved has been comprehensively characterized at the molecular level (68, 69). However, many of the molecules and genes that play a leading role in the suicide and murder of tail muscle cells and the collapse of the notochord remain unidentified. Although apoptosis-related proteins such as caspases are generally considered to contribute to tail resorption, the specific gene expression necessary for the suicide induction of tail muscle cells during spontaneous metamorphosis is unknown. Several ECM-degrading enzymes can cleave collagens, elastin, and other ECM molecules, but no study thus far has identified an enzyme essential for the murder of tail muscle cells, and the possibility remains that multiple enzymes complement each other in this process. Although MMP13 has emerged as a favorable candidate for driving notochord collapse, *MMP13* mRNA is accumulated in the anterior part of the *TR*β-knockout tadpole tail harboring a healthy notochord; this implies that an activator of MMP13 functions as a trigger of notochord dissolution. Addressing these issues in future studies by performing comprehensive RNAseq analyses and using genomic-editing methods to create knockouts of genes of interest will lead to the clarification of the entire mechanism of tail resorption.

## Author Contributions

The author confirms being the sole contributor of this work and has approved it for publication.

### Conflict of Interest Statement

The author declares that the research was conducted in the absence of any commercial or financial relationships that could be construed as a potential conflict of interest.
